# Progress in Dermatology

**Published:** 1896-08-08

**Authors:** 


### Progress in Dermatology.

**Eczema.**—Bowen,[1] in discussing the treatment of eczema, recommends that arsenic should never be used as a routine treatment, especially in acute cases, but that it should be reserved for chronic cases which do not yield to local treatment. He points out that any ointment made with fat is likely to cause irritation by the splitting up of the fat into a fatty acid and glycerine, and he therefore recommends that vaseline should be used as the base of an ointment. He then refers favourably to Lassar's paste, which consists of equal parts of zinc oxide and starch in double the quantity of vaseline; to Pick's gelatine dressing (made by dissolving 30 grains of gelatine and 25 grains of glycerine in 75 grains of water); and to Unna's plaster muslins.

Lassar[2] draws attention to the influence of micro-organisms on the spread of eczema, and insists on the importance of treating the local causes of the disease. He deprecates the practice of considering eczema as a result of internal irritation. For treatment he places great reliance on the use of a bath containing tar, followed by the application of a greasy ointment, since in all cases of any duration the poison has been carried into the lymph lacunæ. He summarises his treatment as follows: (1) Bathing, to remove all products of decomposition; (2) painting the surface with tar or the application of salicylic paste, &c.; and (3) the copious use of Venetian talc powder over any of the previous applications.

Savill[3] describes an outbreak of peri-oral eczema in an East-end Board school. After carefully studying the disease and careful statistics of its outbreak, he considers that it is a definite contagious disease. Its chief clinical characters are: (1) The distribution round the mouth; (2) its uniformity of appearance as red dry, scurfy patches; (3) the fairly uniform course and duration of each patch; and (4) it is practically confined to children under 14 years of age, and occurs only during the summer months. The condition has usually been described as soap rash or seborrhœa sicca. Nearly half the children attending the school were affected during the epidemic, so the author is inclined to consider the disease contagious, and suggests that infection is probably conveyed by the hands of the children. Bacteriological examination showed, in all the cultures, a slowly growing aërobic organism of low vitality, easily stifled by staphylococci, with but little liking for agar, and not liquifying gelatine; the colonies, too, are so minute as easily to be overlooked; they differ from the microbe found in epidemic skin disease.

Copeman,[4] in a report to the Local Government Board, shows that an outbreak of epidemic skin disease in Enfield Workhouse was confined to the inmates who were drinking milk. The disease appeared just after the milk supply had been provided by a new contractor, and another infirmary supplied by the same contractor was also the scene of a similar epidemic at the same time. It is therefore suggested that the epidemic was induced by the milk supply. An enfoliative disorder, similar to epidemic skin disease is known to affect cattle, so the possibility of contracting the disease from animals must be admitted.

Parker[5] relates the case of a woman, aged 65, who was treated for eczema of the legs by thyroid feeding. The treatment was adopted on account of a dryness of the skin and a baggy myxœdematous swelling under the chin, although there were no definite symptoms of myxœdema. The eczema of the legs quickly cleared up, and the dryness of the skin disappeared under thyroid feeding.

**Pemphigus.**—Whipham[6] records two cases of acute pemphigus, one of which occurred in a child, aged two years, and was fatal; the other in a woman, aged 38 years, and recovered. A careful account is given of the necropsy and bacteriology of the fatal case. Fluid from the bullæ and from the lungs was examined, and the following micro-organisms were found: (1) A sarcina, resembling the sarcina lutea; (2) a small slowly-growing sarcina; and (3) a diplococcus. This latter is regarded as the specific organism of the disease, and all the animals inoculated with it died within from four to eight days, the diplococcus being recovered from the heart's blood in each instance. In one of the cases there was a history of a poisoned wound of the finger one week before, and in the other a scratch of a cat occurred five weeks before the onset of the pemphigus. It is therefore probable that a micro-organism was introduced by the injury in each case.

Pernet and Bullock[7] describe eight cases of acute pemphigus occurring in journeymen butchers, in four of which a poisoned wound of the hand had existed before the eruption; out of nine cases, occurring in other than butchers, the patients' occupation brought them in contact with animals or animal products. From bacteriological experiments, the authors conclude that a diplococcus is the cause of the disease; they also consider that the eruption arises as the result of inoculation of the micro-organism into some wound of the hand.

Petrini di Galatz[8] describes three varieties of pemphigus: (1) Pemphigus vulgaris chronica; (2) pemphigus foliaceous; and (3) pemphigus vegetans vulgaris; the two latter forms usually commence as No. 1 and then pass into either of the two types. He does not include under the term of pemphigus those bullous eruptions which disappear in the course of a few weeks, such as hydroa, dermatitis herpetiformes, &c., because he considers that true pemphigus is one of the most serious of dermatoses, and has a very high mortality, while hydroa is one of the least dangerous of all skin diseases.

**Psoriasis.**—Baird[9] insists on the non-parasitic nature of psoriasis, but thinks that its hereditary nature is well marked. He finds that the following treatment gives remarkably good results: An ointment containing 20 grains of salicylate of soda to an ounce of vaseline is applied till the scales have fallen off, then a zinc oxide ointment is used for a few days to reduce the inflammation, after which the following ointment is applied daily after a hot bath: R. salicylate of soda, chrysarobin a.a. gr. 15, oxide of zinc gr. 20, vaseline one ounce, M. He thinks that severe cases of the disease respond more readily to local treatment when thyroid extract is given at the same time. Darier[10] describes two cases of psoriasis limited to the palm of one hand, no other region of the body being affected. The condition is uncommon, and of course may be confused with palmar syphilis; it can, however, be diagnosed by the extreme redness of the eruption, the thinness of the scales, the circinate margin, the usual involvement of the wrist and backs of the hands, and the possible changes in the nails. It is very refractory to treatment, but recovery is ultimately obtained. Thibierge[11] reports the result of thyroid feeding in eleven cases of psoriasis; out of these eight cases were ameliorated but not cured. He regards the method as one only to be resorted to after the failure of all others, and then with due reserve and precautions.

**Lupus.**—Schultze[12] is strongly in favour of the operation of free excision in cases of lupus, and considers that the operation may be successfully performed in even extensive cases of lupus of the face, since the deformity left after excision can nearly always be remedied by Thiersch's grafts without having resource to any plastic operation. In some cases two or three operations are necessary to ensure a perfect result, but the method is as easy in application in the case of recurrences as is treatment by the actual cautery or by means of corrosive ointments.

Derville[13] describes a new method of treating isolated lupus nodules, which he names dilaceration. A scarificator is introduced into the centre of the nodule until it is arrested by cicatricial tissue; it is then forcibly rotated, and some of the tubercular tissue is torn away. A small crystal of chloride of zinc is put into the cavity, a black crust forms which drops off after ten to fifteen days, leaving only a reddish mark. The method is not painful, gives rapid results, and does not interfere with the patient's occupation; it, however, leaves cicatrices, which are often irregular and prominent.

Zerenini[14] has had successful results from the treatment of lupus by local applications of creosote. The sore is first scarified, and a piece of gauze soaked in a solution of creosote in oil or in glycerine is applied, and is kept constantly wet. All the cases showed great improvement, and the author states that this treatment is less apt to be followed by recurrence than curetting or plastic operations.

**Leprosy.**—Reynolds[15] gives a minute description of a case of anæsthetic and macular leprosy which occurred in a man, aged 52, who had not been out of

England for five years. A year before, he had noticed loss of power in the left hand, followed, after a short interval, by numbness of the feet and of the right hand. He then developed, on the chest and back, circles varying in size from one and a half to four inches in diameter, the margins of which were sharply defined, and the skin inside much whiter than normal. The patient also had a perforating ulcer on the right foot.

Sibley[16] describes a boy, aged 16, who was born in Burma, but who had been in England for the last four years. Two years before he had noticed numbness and loss of sensation in the left leg, and he then developed a sore over the great toe. The external popliteal nerve was enlarged, and there was wasting of the calf muscles. The author considers that the case was one of anæsthetic leprosy.

**Urticaria.**—Wright[17] reports two cases of urticaria treated by the administration of calcium chloride, with the view of increasing the coagulability of the blood. He considers that urticaria—the eruption of which is due to the serous hæmorrhages into the skin—is most likely to occur in persons whose blood coagulability is diminished, so he hopes to control the transudation by correcting the defect in blood coagulation. The dose at first was 30 grains, but this was reduced to 20 grains the following day, and to 5 grains at the end of ten days. The itching was quickly relieved, and no further eruption occurred. In one of the cases the urticaria followed an inoculation with antitoxic serum for diphtheria.

Morain[18] recommends the following treatment for urticaria: Tincture of belladonna in doses of from 5 to 15 minims, or quinine in doses of 10 grains should be given by the mouth, while locally, instead of a bath, the surface of the body should be sponged with very hot camomile decoction containing 1 per cent. of carbolic acid. When the skin has been dried the following ointment should be applied: Carbolic acid, 8 to 15 grains; zinc oxide, lanoline, and vaseline a.a. 300 grains, M. The whole of the body and the bedclothes should be freely dusted with starch.

Meynet[19] recommends friction with salt for urticaria The wheals are moistened with cold water and kitchen salt is rubbed in for ten to fifteen seconds. A slight burning, followed by a pleasant sensation of coolness, is felt; then the itching decreases or ceases entirely and the papules disappear. Zinc ointment or rice powder may then be applied.

**Scleroderma.**—Dercum[20] describes three cases of scleroderma in which the starting point was connected with an impression on the nervous system; in one instance an injury to the head, in another repeated chilling of the surface from constantly entering an icehouse, seemed to be the important factor in determining the disease. In one case, in which the finger joints of one hand were stiff, the Röntgen ray photography showed that the shade of the bones was continuous across those joints which were immovable. Thyroid feeding was used in one case, but this treatment seemed to hasten the disappearance of the fat. Dana[21] mentions the association of scleroderma with facial hemi-atrophy, of which he refers to several cases. White[22] describes a case of scleroderma which occurred in a woman, aged 44, who was also affected with osteoarthritic changes in many large joints. She was also subject to frequently-repeated syncopal attacks in the fingers and hands. These would come on twenty to thirty times a day after the least exposure to cold. During the previous three years she had suffered from a frequently-recurring eruption of heat bumps on the forearms and legs, and the skin had gradually become hardened. The author suggests that scleroderma, which is a simple overgrowth of the fibrous tissue of the skin, may be due to chronic inflammation resulting from intermittent blood supply. With regard to treatment, he recommends warmth and galvanism locally, while internally he gives drugs which produce arterial dilatation, amongst which he mentions jaborandi, salicylates, and thyroid feeding.

Weber[23] relates a case where strings of scleroderma had existed for three years on the left upper arm and wrist; they finally extended to the left of the middle line. There was no contraction of the skin, and the senses of touch and of pain were unaffected; some rheumatoid pains were complained of. No improvement followed the use of arsenic or of salicylates, but after nine months' treatment with thyroid feeding the disease was practically cured.

**Parasitic Diseases.**—Jullien and Descouleurs[24] call attention to a non-sulphurous treatment of the itch, their conclusions being based on 300 cases. An acarus will remain alive for sixteen hours in sulphur fumes, and for one hour when immersed in flowers of sulphur or in ointment, while it cannot survive more than twenty minutes when placed in contact with Balsam of Peru; this latter drug also kills the eggs. The treatment recommended is very simple. Before going to bed the Balsam of Peru is painted over the body and rubbed in with the hand; in the morning a hot bath is taken, and the cure is said to be complete. The method is specially indicated when scabies is complicated with impetigo, eczema, ecthyma and boils, or in feeble subjects and those with delicate skins.

Roberts[25] states that scabies may be derived from the horse, and that when so contracted it is capable of being cured more readily than when communicated by man, since the acarus of the horse does not burrow so deeply. With regard to treatment, he advises the vigorous soaping and scrubbing with a nail brush of the parts which the acari effect, followed by inunction with an antiseptic ointment. This inunction is repeated night and morning for three days, after which there should not be a single living acarus present; attention must then be directed to the treatment of the nervous and toxic symptoms, and the local disease, which is then either eczema or dermatitis, must be treated on soothing lines.

Stelwagon,[26] in a clinical lecture on Barber's itch, states that the disease is due to the same fungus as ordinary ringworm, and so suggests that its name should be tinea trichophytina barbæ. With regard to treatment, the most essential part consists in free depilation, followed by the application of any of the so-called parasiticides; of these, he prefers either a sulphur or mercurial line of treatment. In the sulphur treatment, a lotion of sodium hyposulphite (one drachm to the ounce), and a 10 to 20 per cent. ointment of præcipitated sulphur, are conjointly prescribed; in the mercurial treatment he uses a corrosive sublimate lotion ($\frac{1}{2}$ to 2 grains to the ounce) together with an ointment containing either 10 per cent. of oleate of mercury, or white precipitate, 30 grains to the ounce. Whichever treatment is chosen, first the lotion is thoroughly applied to the diseased and adjacent parts and allowed to dry, then the ointment is rubbed in. This should be done twice daily till all signs of the disease have disappeared, and then intermittently for several weeks.

Ehrmann[27] relates a case of sycosis scleroticans, which he had treated successfully by electrolysis. This disease commences as a small boil, and usually affects the nape of the neck, creeping upwards into the scalp. When applying electrolysis the needle should be retained deeply plunged into the tissues for a longer time than is customary when removing ordinary hairs.

**Acne.**—Bronson,[28] in opening a discussion of acne, considers the bacteriology of the disease as well as its clinical features and treatment. The following are the chief indications for treatment: (1) Reduction of the hyperkeratosis; (2) removal of comedones; and (3) disinfection of the follicles and antiphlogistic measures. To remove the comedones, the tips of pustules and the corneous elevations, he uses a special currette, which is swept over the affected area after the skin has been put on the stretch. When the comedones cannot be easily removed he applies a lotion of vinegar two parts, glycerine three parts, and koalin four parts, which softens them and makes their extraction easy. In acne indurata it is necessary to incise the nodules and currette them deeply; but the whole of them cannot often be done at one sitting. Immediately after the curretting and extraction of comedones the skin should be well bathed with a 1 in 1,000 solution of bichloride of mercury; the patient should then use daily an ordinary sulphur lotion, or a more elegant one containing 3 to 4 per cent. of resorcin in eau de cologne and water.

Carrier[29] describes a case of atrophic comedone in a boy eleven years of age. The disease commenced eight years previously, and had gradually spread over the whole of one side of the face, which was now as badly pitted as if the child had suffered from small-pox. The lesions resembled comedones, but the plugs could not be squeezed out, and could only be removed with a needle; these comedones gradually increased in size, and then slowly disappeared without inflaming, and left large pits, the result of the destruction of the sebaceous glands. The hard plug is composed mostly of epithelial scales, and causes, by pressure, atrophy of the sweat gland. As regards treatment, he recommends that the comedones should be softened by washing with soap and fats, and should then be picked out with a needle, 10 to 20 being done at each sitting; sulphur or resorcin ointment should then be applied. The scarring, of course, is permanent.

**Diseases of the Hair.**—Fournier[30] observes that there are two important causes of baldness which are little considered. The first is abuse of water, and the second deficiency or absence of oil. He is of opinion that most bald people would have been in possession of a luxurious chevelure had they applied some fatty matter to the scalp from time to time. Although some men frequently apply grease to the hair they smooth it afterwards with a wet brush, by which water tracts down the hair and wets the roots; excessive perspiration also causes baldness by keeping the roots wet. Nothing cleans children's heads so well as the yolk of an egg, the action of which is favoured by the existence in it of an emulsive principle. For adults the employment of pomade, or some other oily substance, should be part of their daily toilet.

Féré[31] relates two cases of alopecia areata occurring in epileptics, in which a neurotic, rather than a parasitic, origin seemed the more probable. In both cases the hair suddenly fell out in circular patches within a few days of a severe fit, or series of fits, which had occurred after a considerable period of freedom. The hairs round the bald patches were fine and not broken. The hair returned without any treatment.

[1] Ther. Gaz., Jan. 15, 18[illegible]6, p. 37. [2] Brit. Journ. of Derm., April, 1896, p. 155. [3] Lancet, Jan. 4, 1896, p. 9. [4] Brit. Med. Journ., May 2, 1896. [5] Brit. Med. Journ., April 18, 1896. [6] Lancet, May 2, 1896. [7] Brit. Journ. Derm., May, 1896, p. 157; and Brit. Journ. Derm., June, 1896, p. 205, [8] Lancet, April 18, 1896. [9] Tri. State Med. Journ., Feb., 1896, p. 43. [10] Med. Week., April 24, 1896, p. 199. [11] Ed. Med. Journ., May, 1896, p. 1,070. [12] Practitioner, Feb., 1896, p. 216. [13] New York Med. Journ., Feb. 22, 1896. [14] Ther. Gaz., Feb 15, 1896, p. 111. [15] Brit. Med. Journ., Feb. 8, 1896, p. 337. [16] Brit. Med. Journ., March 14, 1896, p 659, [17] Brit. Journ. of Derm., March, 1896. p. 82. [18] New York Med. Journ., Feb. 1, 1896, p. 163. [19] Med. Rec., Feb. 22, 1896. [20] New York Med. Rec., April 25, 1896, p. 603. [21] New York Med. Rec., April 25, 1896, p. 605. [22] Lancet April 25, 1896. [23] Med. Week, Feb 21, 1896, p. 91. [24] Lancet, April 18, 1896. [25] Int. Med. Mag., Mar., 1896, p. 134. [26] Int. Med. Mag., Feb., 1896, p. 40. [27] Ed. Med. Journ., Mar., 1896, p. 870. [28] New York Med. Journ., Mar. 28, 1896, p. 401, 422. [29] Int. Clin., vol. iv., p. 348. [30] Ed. Med. Journ., April, 1896, p. 974. [31] Ed. Med. Journ., March, 1896, p. 867.

